# Effects of Low-level Brodifacoum Exposure on the Feline Immune Response

**DOI:** 10.1038/s41598-018-26558-3

**Published:** 2018-05-25

**Authors:** Jennifer H. Kopanke, Katherine E. Horak, Esther Musselman, Craig A. Miller, Kristine Bennett, Christine S. Olver, Steven F. Volker, Sue VandeWoude, Sarah N. Bevins

**Affiliations:** 1 Colorado State University, Department of Microbiology, Immunology, and Pathology, Fort Collins, 80523 USA; 2 USDA-APHIS, National Wildlife Research Center, Fort Collins, 80521 USA

## Abstract

Anticoagulant rodenticides have been implicated as a potential inciting factor in the development of mange in wild felids, but a causative association between anticoagulant rodenticide exposure and immune suppression has not been established. Specific-pathogen-free domestic cats were exposed to brodifacoum over a 6-week period to determine whether chronic, low-level exposure altered the feline immune response. Cats were vaccinated with irrelevant antigens at different points during the course of the experiment to assess recall and direct immune responses. Measures of immune response included delayed-type hypersensitivity tests and cell proliferation assays. IgE and antigen-specific antibodies were quantified via ELISA assays, and cytokine induction following exposure to vaccine antigens was also analyzed. While cats had marked levels of brodifacoum present in blood during the study, no cats developed coagulopathies or hematologic abnormalities. Brodifacoum-exposed cats had transient, statistically significant decreases in the production of certain cytokines, but all other measures of immune function remained unaffected throughout the study period. This study indicates that cats may be more resistant to clinical effects of brodifacoum exposure than other species and suggests that the gross impacts of environmentally realistic brodifacoum exposure on humoral and cell-mediated immunity against foreign antigen exposures in domestic cats are minimal.

## Introduction

Anticoagulant rodenticide baits are an important pest control tool, but they also pose a health threat to non-target species. Upper trophic-level predators, including raptors, bobcats, coyotes, mountain lions, and other rodent-eating species, are often exposed to rodenticides by consuming poisoned rodents^1–7^. In Southern California, blood and liver samples from 64 bobcats (*Lynx rufus*) revealed that 92% had been exposed to anticoagulant rodenticides^8^.

Anticoagulant rodenticides interrupt blood clotting by inhibiting vitamin K epoxide reductase, an enzyme that is essential for the conversion of vitamin K into its active form^9^. The active form of vitamin K is necessary for the synthesis of various components of the coagulation cascade, including clotting factors II, VII, IX, and X^9^. Sufficient exposure to anticoagulant rodenticides can result in profound bleeding and death.

First-generation anticoagulant rodenticides such as warfarin have shorter half-lives and generally require repeated dosing to produce toxicity, while second-generation anticoagulant rodenticides can be toxic with a single dose^10,11^. Widely used second-generation anticoagulant rodenticides include brodifacoum, bromadiolone, and difenacoum^10^. Second-generation rodenticides are lipophilic and bioaccumulate in liver, so non-target wildlife may develop toxicity following consumption of multiple, individually non-toxic doses over time^11^.

Exposure to these first and second generation anticoagulant rodenticides has been documented in bobcat and mountain lion (*Puma concolor*) populations in California, which have also been increasingly debilitated by severe notoedric mange (feline scabies). Recent studies have suggested that this is attributable to chronic second-generation anticoagulant rodenticide exposure based on correlative evidence showing that non-domestic felids with severe mange have also been exposed to anticoagulant rodenticides^8,11,12^, and that urban bobcats with evidence of rodenticide exposure have perturbations in gene expression^13^, white blood cell counts^14^, and serum biochemistry parameters^14^. While the causative agent of feline scabies – *Notoedres cati* – generally causes a transient skin infestation, immunocompromised animals may develop generalized, crusting mange^15–17^. This more severe form of the disease may be due to an ineffective Th2 immune response and can result in debilitation and death^16^.

In one study, post-mortem examination of mange-infested bobcats revealed variable levels of anticoagulant rodenticide residues present in the livers of all bobcats tested, in addition to various hematologic and serum biochemistry abnormalities^12^. Some of these bobcats were also tested for select infectious diseases (FIV, FeLV, FIP, leptospirosis) and test results were predominantly negative^12^, indicating that any potential immune suppression is likely caused by something other than the most common feline immune-suppressing pathogens^17^. Therefore, it has been speculated that chronic exposure to second-generation anticoagulant rodenticides has immunomodulatory effects in felids, resulting in severe – and sometimes fatal – cases of notoedric mange in California bobcats and pumas, in addition to broader immune dysfunction in these species^13,14^. While domestic cats (*Felis catus*) are remarkably resilient to the anticoagulant effects of rodenticides compared to other species^18^, the effects of sub-toxic anticoagulant rodenticide exposure in cats have not been fully explored.

Beyond its role in coagulation, vitamin K and its derivatives seem to play a role in diminishing the production of inflammatory cytokines *in vitro*^19,20^. It is conceivable that non-lethal rodenticide exposure could interfere with vitamin K synthesis at a sub-clinical level, subsequently disrupting the balance of inflammatory and anti-inflammatory cytokines and producing immune dysfunction. Moreover, in rodents, chronic exposure to warfarin–a first-generation anticoagulant–causes a pro-inflammatory response in circulating granulocytes and affects IL-6 and TNF-α production^21,22^. However, a closely related drug, acenocoumarol, diminishes Th1 immune responses and pro-inflammatory cytokines in human cells *in vitro*^23^. These studies and others indicate that anticoagulants may modulate various aspects of immunity through vitamin K-related mechanisms as well as associated peripheral pathways.

Here, we use a specific-pathogen-free (SPF) domestic cat model to examine the effect of repeated, sub-toxic anticoagulant rodenticide exposure on the immune responses of felids.

## Results

### Experimental brodifacoum exposure has minimal gross effects on feline health

**Figure 1:**
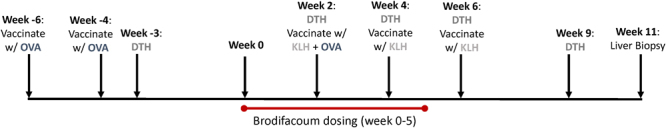
Experimental timeline.

To model an environmentally realistic, chronic exposure scenario, cats in the brodifacoum-treated group (n = 5) were fed 0.05 mg/kg body weight brodifacoum mixed into canned cat food once weekly for six consecutive weeks (weeks 0–5) (Fig. 1). This dose was selected based on the findings of a conservation-based aerial baiting operation, where animals that directly consumed brodifacoum bait were found to contain approximately 1 µg brodifacoum/g meat^24^. Thus, a 4 kg cat consuming one poisoned rat (~200 g) would ingest a dose of 0.05 mg/kg brodifacoum. Using this estimate, we fed cats in the brodifacoum-treated group the equivalent of one brodifacoum-intoxicated rodent per week to model a realistic dose and frequency of brodifacoum exposure. Cats in the control group (n = 5) received an equivalent amount of inert bait. Group size was determined based on the minimum number of animals needed to permit discrimination of dose effects on hematocrit and clotting time based upon previous studies of brodifacoum in non-target species^25^.

We monitored brodifacoum-exposed and control cats for symptoms of brodifacoum intoxication (lethargy, tachycardia, tachypnea, anemia, coagulopathy, hemorrhage) throughout the study. We also tracked the cats’ weights and rectal temperatures as additional measures of health, and these were consistently within normal ranges in both control and brodifacoum-treated cats.

**Figure 2:**
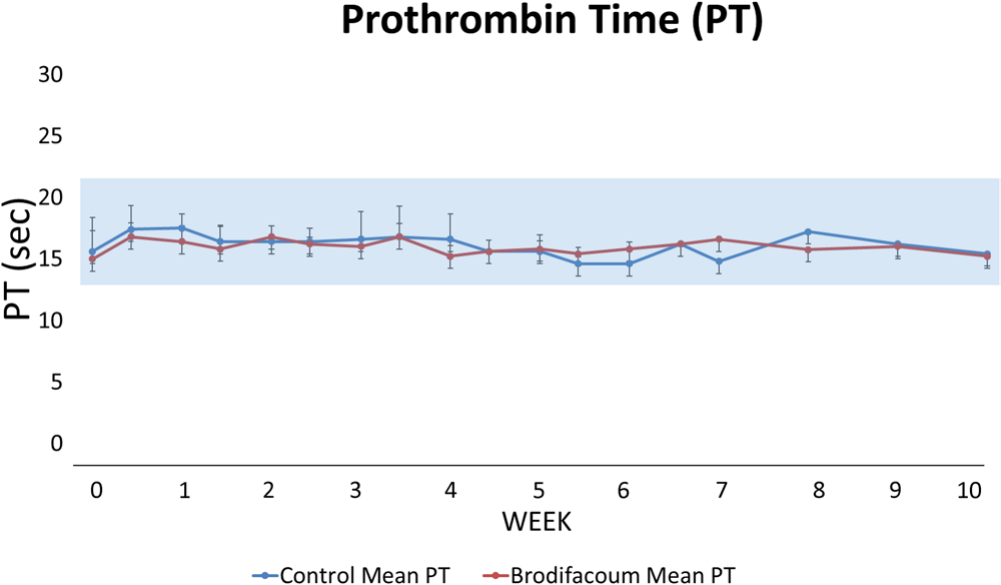
Mean prothrombin times (PT) are normal in brodifacoum-treated cats. Shaded area represents normal PT range for healthy cats. Standard deviation for each group shown at time points.

Complete blood counts (CBC) and prothrombin times (PT) were regularly monitored to assess key hematologic and immunologic parameters. CBC monitoring included: hematocrit, platelet count, nucleated cell count, neutrophil %, lymphocyte %, monocyte %, eosinophil %, neutrophil count, lymphocyte count, monocyte count, and eosinophil count. No significant changes occurred in the CBCs of brodifacoum-treated or sham-treated cats at any time point. PT and blood clot formation also remained normal for all cats, regardless of exposure to brodifacoum (Fig. 2).

Additional assays measuring vitamin K-dependent clotting pathways were performed to identify subtle changes in clotting not detectable by PT. Proteins Induced by Vitamin K Antagonism or Absence (PIVKA) were measured using a commercially available human quantitative sandwich enzyme immunoassay. Pre-exposure (week 0) and post-exposure (week 9) plasma from brodifacoum-treated and sham-treated cats and negative control samples were below the limit of detection at both time points. Similarly, no differences in factor X activity were detected between treated and untreated cats at weeks 0 and 9 using a commercial chromogen-based factor X quantification kit.

### Cats have marked levels of liver brodifacoum following chronic exposure

**Figure 3:**
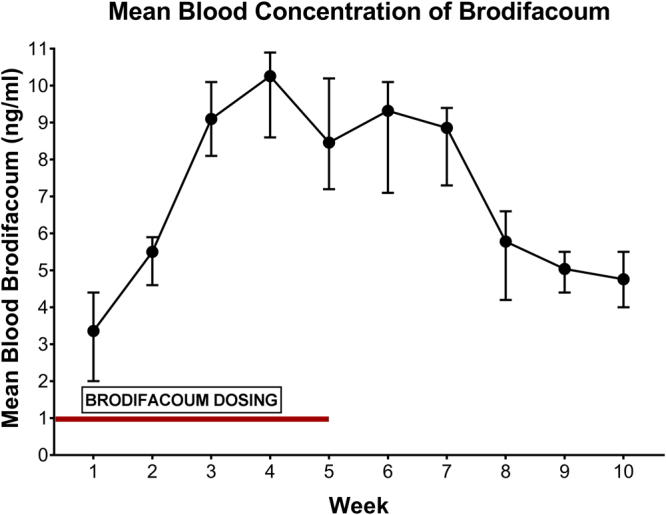
Mean blood brodifacoum levels peak 3–4 weeks following start of dosing. Range of values shown at each time point. Initial dose of brodifacoum started 7 days prior to initial data point, on week 0. Time frame of brodifacoum dosing depicted with red line (weeks 0–5).

Using high-performance liquid chromatography combined with atmospheric pressure chemical ionization and tandem mass spectrometry (HPLC-APCI-MS/MS), we measured the concentration of brodifacoum present in whole blood samples collected during and following brodifacoum exposure. Brodifacoum concentrations peaked after 5 weeks (week 4, mean: 0.0103 µg/ml, range: 0.0086–0.0109 µg/ml) despite ongoing exposure, and then gradually decreased over subsequent weeks (Fig. 3). At the final blood collection, mean brodifacoum levels had decreased to 0.0048 µg/ml (week 10, range: 0.0040–0.0055 µg/ml) (Fig. 3).

Six weeks after the final bait exposure (week 11), the brodifacoum-treated group had mean liver brodifacoum levels of 1.806 µg/g (SE: 0.473 µg/g, range: 1.670–1.940 µg/g). Cats in the brodifacoum-treated group did not exhibit any clinical signs of brodifacoum intoxication (elevated PT, altered PIVKA and factor X activity, hemorrhage, anemia), despite the marked levels of brodifacoum detected in all liver samples. Brodifacoum was not detected in the blood or livers of control cats.

### Brodifacoum-treated and control cats have similar delayed-type hypersensitivity (DTH) responses

While cats did not develop overt hematologic or clotting abnormalities in response to brodifacoum exposure, we wanted to examine whether brodifacoum-treated cats could mount a normal immune response. We vaccinated treated and non-treated cats with the irrelevant antigens keyhole limpet hemocyanin (KLH) and ovalbumin (OVA) to evaluate the potential effects of brodifacoum on primary and recall immune responses. Following vaccination, cell-mediated immunity to OVA and KLH were tested with delayed-type hypersensitivity (DTH) tests to determine if anticoagulant-altered immune responses were manifesting in the dermis, as would potentially be the case with severe mange reactions.

**Figure 4:**
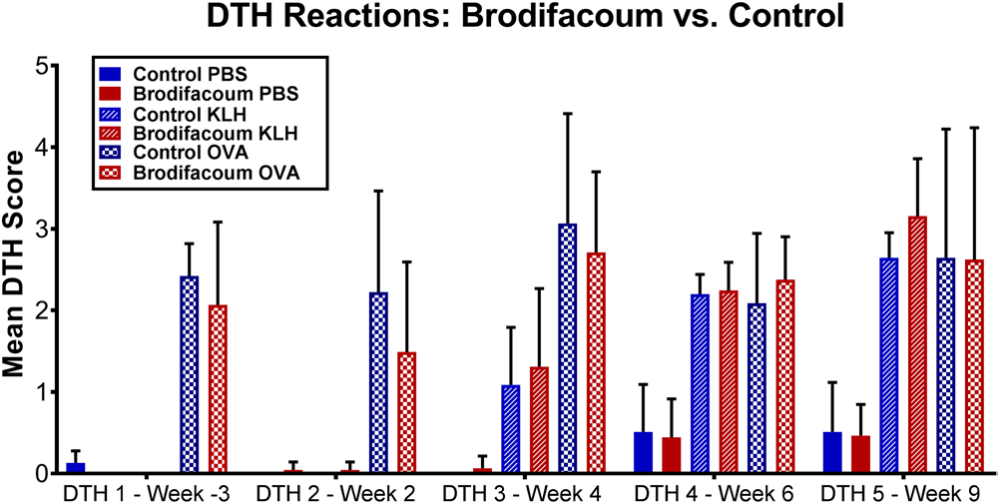
Brodifacoum- and sham-treated cats have similar delayed-type hypersensitivity (DTH) responses. Mean DTH score (scale: 0–5) for cats in brodifacoum and sham bait treated groups. PBS = vehicle control. Ovalbumin (OVA) concentration decreased to 0.25 mg/ml on weeks 6 and 9.

Vaccinated cats in both the brodifacoum-treated and sham-treated groups developed strong positive reactions (redness and induration) to intradermal injections of OVA and KLH following initial vaccinations, and KLH and OVA DTH reactions became increasingly prominent with repeated vaccinations (Fig. 4). However, no significant differences in DTH response were detected between groups at any time point, indicating that cell-mediated immunity was predominantly normal in brodifacoum-treated cats despite chronic brodifacoum exposure.

### Brodifacoum-treated and control cats have similar antibody responses

**Figure 5:**
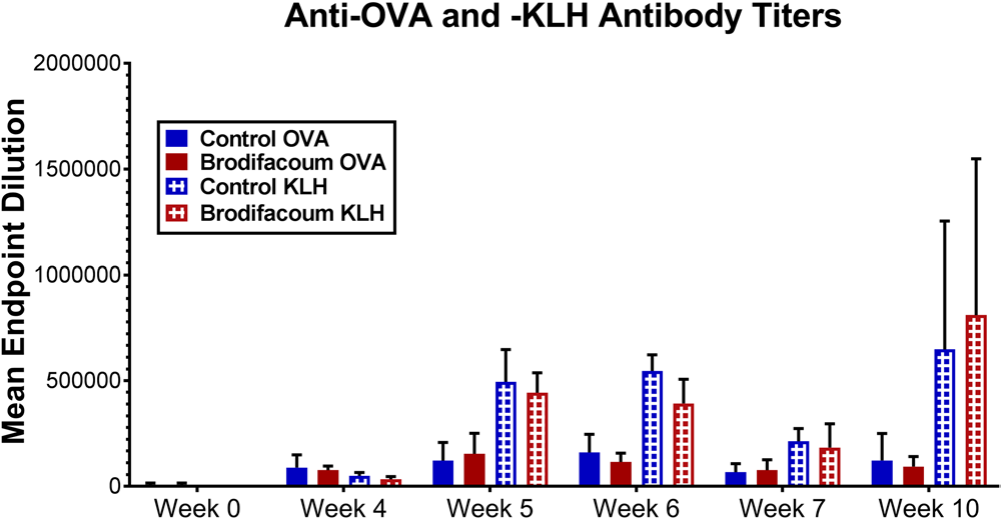
Brodifacoum- and sham-treated cats have similar antibody responses to ovalbumin (OVA) and keyhole limpet hemocyanin (KLH). Mean ELISA endpoint dilution, indicating last positive dilution shown. Standard deviation shown for each group. No significant differences noted between sham- and brodifacoum-treated groups. There was a trend towards lower anti-KLH antibody titers in brodifacoum-treated cats at week 6 (p = 0.17).

We then investigated whether humoral immunity was diminished in brodifacoum-treated animals. To quantify antibodies present against OVA and KLH, we performed ELISA tests on serum collected from cats in both groups during and following bait exposure. There were no significant differences in concentration of serum antibodies to OVA between the brodifacoum-treated and sham-treated group at any time point (Fig. 5). At week 6, there was a trend of lower anti-KLH antibody titers in cats in the brodifacoum-treated group compared to the sham-treated cats (p = 0.17), while antibodies to KLH were otherwise the same between groups at all other time points.

We then specifically measured serum IgE concentrations. As IgE is a key modulator of immunity to parasites, we speculated that brodifacoum might cause perturbations in IgE production, resulting in impaired immunity to external parasitism. However, serum IgE concentrations were not significantly affected by brodifacoum exposure. Cats in the control group had significantly higher levels of IgE than brodifacoum-treated cats, but this difference was present throughout the study – even before brodifacoum exposure – suggesting that individual outliers may have had a large effect (see Supplementary Fig. S1).

### Brodifacoum exposure does not alter cell proliferation responses

**Figure 6:**
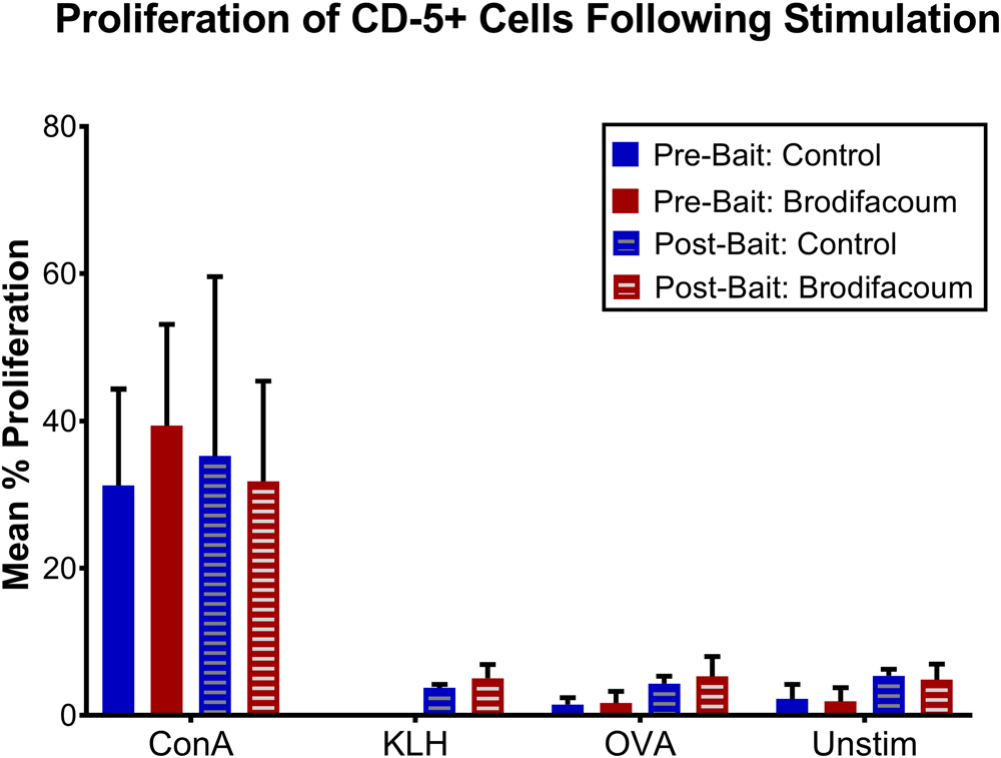
Brodifacoum- and sham-treated cats have similar cell proliferations responses. Mean percent proliferation of CD-5+ cells in response to concanavalin A (ConA), ovalbumin (OVA), keyhole limpet hemocyanin (KLH), or unstimulated cells measured by incorporation of 5-ethynyl-2′-deoxyuridine (EdU) via flow cytometry. Standard deviation for each group is shown. Pre-bait exposure CD-5+ cells were collected on week 0 (day 0) prior to first brodifacoum treatment. Post-bait exposure CD-5+ cells were collected on week 10 (day 70). Cats had not been vaccinated with KLH prior to the first cell proliferation assay, so response to KLH-stimulation was not measured on week 0.

To test the overall immune competence of brodifacoum- and sham-treated cats, we performed cell proliferation assays pre- and post-bait exposure (weeks 0 and 10, respectively). We specifically assayed lymphocyte proliferation by measuring CD5+ cell population expansion in response to stimulation with concanavalin A (ConA), OVA, or KLH. Non-stimulated peripheral blood mononuclear cells (PBMCs) were used as a control. No significant difference in cell proliferation was detected between OVA-stimulated and KLH-stimulated cells collected at week 10 in either the treatment or control group (Fig. 6). Moreover, there were no differences in the cell proliferation responses between treated and untreated cats, regardless of the stimulant used to induce cell proliferation.

### Brodifacoum treatment is associated with alterations in cytokine expression

**Figure 7:**
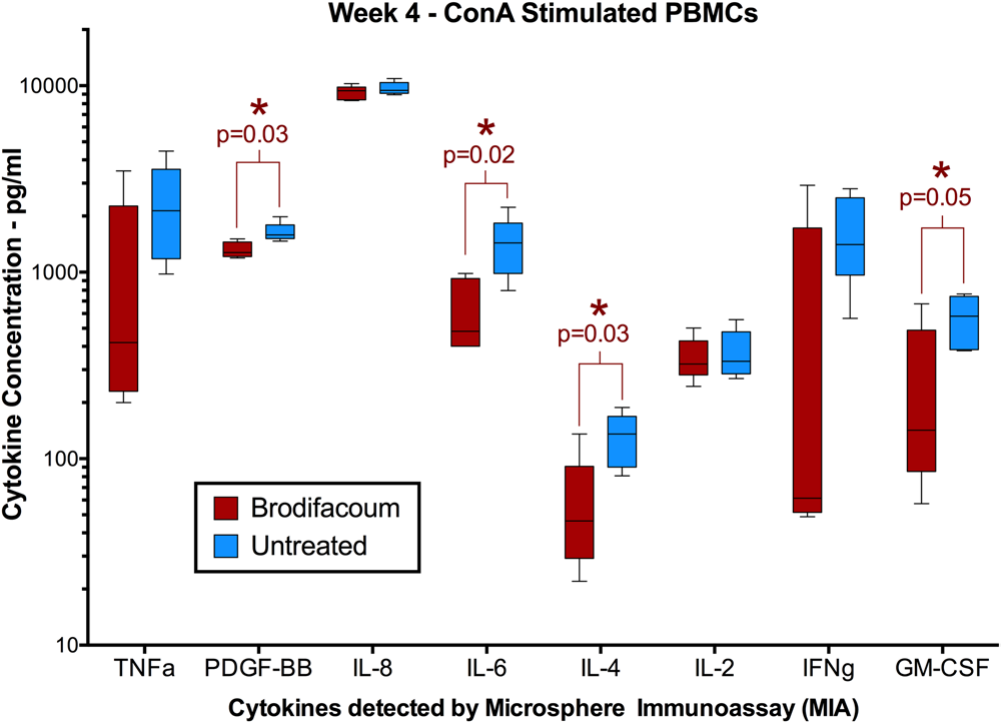
Concanavalin A (ConA)-stimulated peripheral blood mononuclear cells (PBMCs) from brodifacoum-treated cats have significantly lower levels of certain cytokines than sham-treated cats after four weeks of brodifacoum exposure. Box-and-whisker plot shows the median, interquartile, and concentration range of each cytokine in picograms (pg) per milliliter (ml) of PBMC culture medium.

In order to assess innate immunomodulatory effects of brodifacoum exposure, PBMCs were collected from treated and untreated cats on weeks 0, 4, and 6 and subjected to various stimulatory conditions (ConA-, OVA-, KLH-, or sham-stimulated). At each time point, the amount of INFγ, IL-1β, IL-2, IL-8, IL-10, MCP-1, TNFα, Fas, SDF-1, SCF, RANTES, PDGF-BB, KC, IL-18, IL-13, IL-12 (p40), IL-6, IL-4, GM-CSF, and Flt-3 ligand was quantified by microsphere immunoassay to detect differences in cytokine expression between brodifacoum-treated and untreated cats. The most significant effect occurred in ConA-stimulated PBMCs at week 4 (Fig. 7), for which there was a significant decrease in IL-6 (p = 0.02), IL-4 (p = 0.03), GM-CSF (p = 0.05), and PDGF-BB (p = 0.03) expression in brodifacoum-treated cats compared to untreated cats. There was also a trend at this time point for TNFα (p = 0.19), KC (p = 0.12), and IL-18 (p = 0.11) to be decreased in brodifacoum-treated PBMCs following ConA stimulation, but these results were not significant. No significant differences in cytokine expression were detected between brodifacoum-treated and untreated cats at the next tested time point, week 6. When PBMCs from brodifacoum-treated cats were evaluated specifically over time, there was a significant decrease in TNFα (p = 0.001) at week 4 and a significant increase in RANTES (p = 0.05) at week 6 when compared to cytokine levels prior to treatment (week 0).

**Figure 8:**
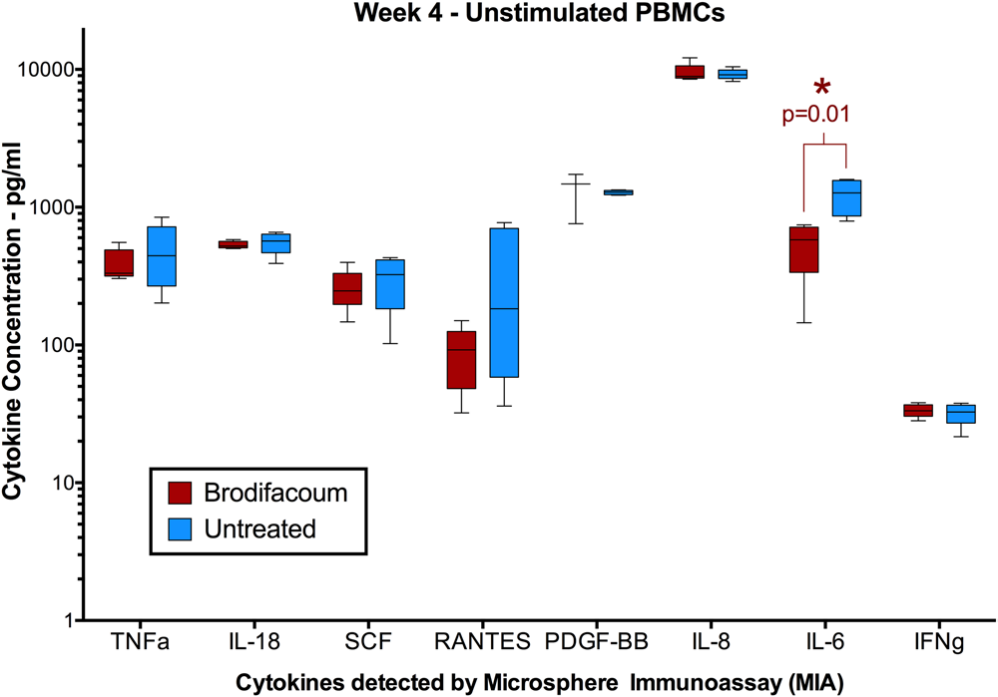
Unstimulated peripheral blood mononuclear cells (PBMCs) from brodifacoum-treated cats have significantly lower levels of IL-6 than sham-treated cats after four weeks of brodifacoum exposure. Box-and-whisker plot shows the median, interquartile, and concentration range of each cytokine in picograms (pg) per milliliter (ml) of PBMC culture medium.

**Figure 9:**
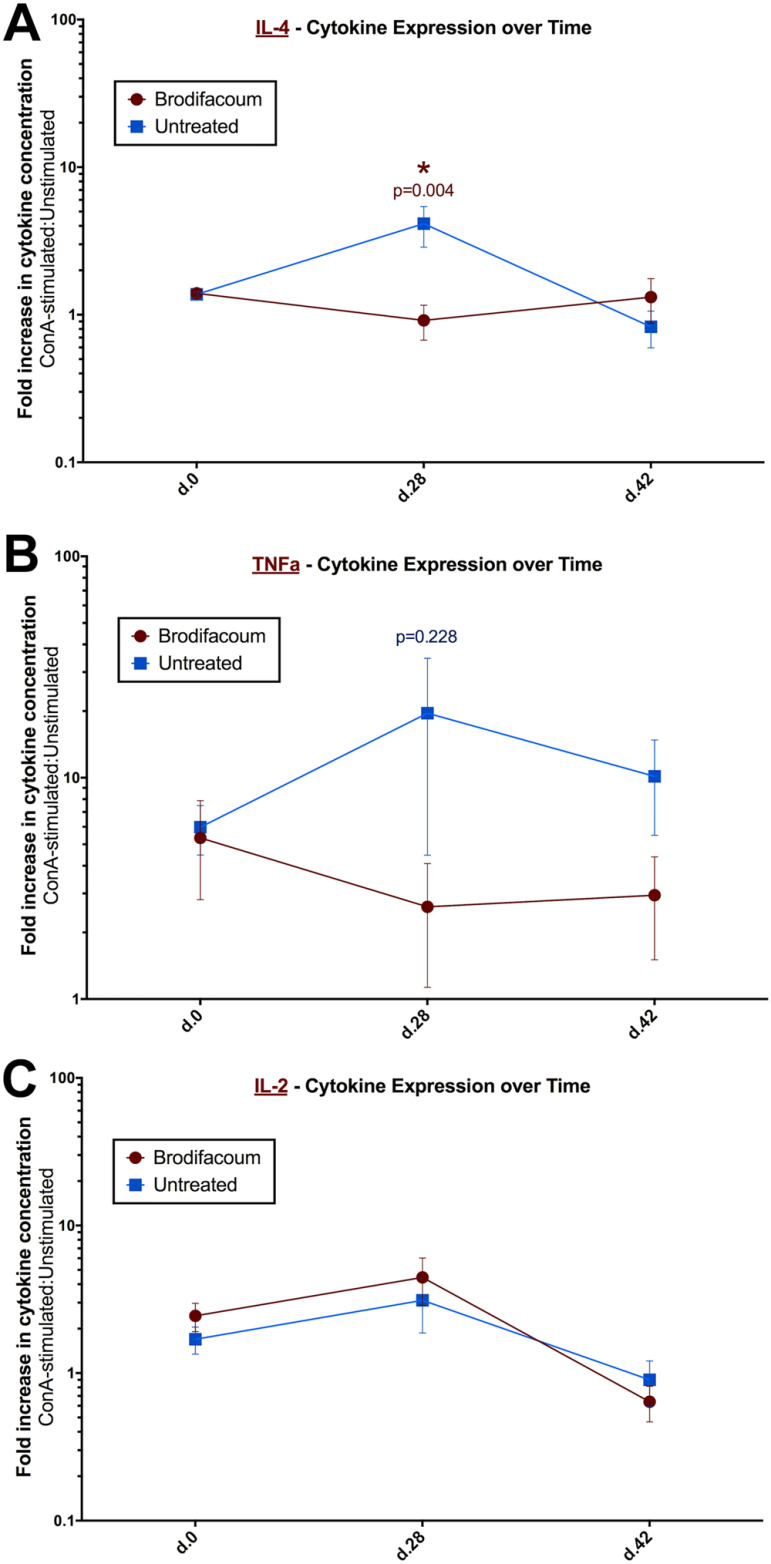
Ratio of cytokine expression in stimulated versus unstimulated peripheral blood mononuclear cells (PBMCs) differs between brodifacoum-treated and control cats. Ratio between concanavalin A (ConA)-stimulated and unstimulated PBMC cytokine expression represented as fold increase in cytokine expression (ConA:unstimulated). Error bars represent standard error of the mean.

Compared to PBMCs from untreated cats, unstimulated PBMCs from brodifacoum-treated cats exhibited significantly decreased IL-6 (p = 0.01) at week 4 (Fig. 8). For analytes with sufficient levels of cytokine expression at all time points (IL-4, TNFα, IL-2), the ratio of cytokine expression in stimulated versus unstimulated PBMCs was calculated (represented as fold increase in cytokine expression – ConA/Unstimulated). For this measurement, we observed that brodifacoum treatment produced a significantly decreased effect in IL-4 expression (p = 0.004) at week 4 (Fig. 9A). Although not statistically significant, there was also as a slightly decreased effect in TNFα expression at week 4 (p = 0.228) (Fig. 9B), while no effect was observed in IL-2 expression (Fig. 9C). Surprisingly, no significant differences in cytokine expression were observed between brodifacoum-treated and untreated cats in OVA- or KLH-stimulated PBMCs at any time point.

## Discussion

Numerous studies have explored the adverse effects of anticoagulant rodenticides on non-target species, but it remains unclear whether sub-toxic doses of these compounds can affect immune responses, as has been suggested for bobcats and mountain lions with severe notoedric mange in California^11–14^. Other studies have detected second-generation anticoagulant rodenticide residues in the livers of non-target species, but have not reported increased incidence of mange or other immune anomalies^26–28^.

Therefore, we designed this study to determine whether cats develop immunological changes in response to chronic, low-level brodifacoum exposure, as might occur in a natural setting where upper-level predators occasionally consume prey with brodifacoum residues. Brodifacoum-treated cats consumed roughly the equivalent of one brodifacoum-intoxicated rodent per week for six consecutive weeks, modelling a realistic exposure scenario. This dose and frequency allowed us to obtain measurable amounts of brodifacoum in the blood and tissues of exposed cats without producing acute coagulopathies as would be seen at higher doses. There are reports of the LD50 being as high as 25 mg/kg for cats^29,30^, while the lowest reported LD50 for brodifacoum in cats is 0.25 mg/kg^31^. Our dose of 0.05 mg/kg/week was 1/5th of the lowest reported toxic dose and 1/500th that of the highest reported toxic dose. The cats in our study showed no hemogram or clotting abnormalities despite blood brodifacoum levels up to 0.0109 µg/mL and liver brodifacoum levels up to 1.940 µg/g. Moreover, specific clotting assays were within normal ranges for brodifacoum-treated cats, indicating that cats had no detectable changes in vitamin K-dependent clotting pathways. These findings confirm that cats are highly resistant to the anticoagulant effects of rodenticides. The physiologic mechanisms underlying this species-specific insensitivity remain unknown and warrant further study.

In a study of bobcats in California, Seryies *et al*. detected a mean concentration of 0.23 ppm of brodifacoum, 0.62 ppm of bromadiolone, and 0.49 ppm of diphacinone in the livers 19 mange-infested bobcats^12^. In an additional study, the authors concluded that severe mange in bobcats was significantly associated with brodifacoum exposure and brodifacoum concentration^8^. Despite these associations, the domestic cats in our study had higher brodifacoum residues in their livers than the mange-infested bobcats (mean: 1.806 ppm, SE: 0.473 ppm), but did not develop striking immunologic derangements. Mange-associated mortality in bobcats was also found to be significantly associated with liver anticoagulant residues > 0.05 ppm^11^, whereas there was no domestic cat mortality in this study despite substantially higher liver residues. It is possible that bobcats and other wild felids are more sensitive to the effects of anticoagulant rodenticides than domestic cats, or that a combination of second generation anticoagulant rodenticides may cause more profound derangements than exposure to a single compound. Alternatively, the afore-mentioned associations between liver anticoagulant residues, mange, and bobcat mortality may be correlative findings, rather than causative.

We monitored several immune system parameters throughout the study to account for a broad range of potential effects. Collectively, our findings demonstrate that the majority of immune system components showed no differential effects between brodifacoum-treated and sham-treated domestic cats. We noted a significant difference in serum IgE concentrations between brodifacoum-treated and control cats, but this effect was present before brodifacoum exposure and appeared to be due to the presence of one or two cats with consistently high IgE in the control group. Importantly, this outlier effect was not apparent in our other analyses, making it unlikely that any results were masked or falsely detected due to the presence of individual outliers.

Low-level brodifacoum exposure did appear to influence certain cytokine levels, but these differences did not manifest clinically and were apparently transient. Differences in interleukin-4 (IL-4) and IL-6 expression – which are important for B-cell proliferation and IgE isotype switching – were detected on week 4, possibly explaining the lower anti-KLH antibody titer in brodifacoum-treated cats on week 6. In contrast to our findings, a recent study comparing anticoagulant rodenticide-exposed and unexposed bobcats found a significant association between rodenticide exposure and expression of keratinocyte chemoattractant (KC), stem cell factor (SCF), and IL-12p40 levels, but no differences in IL-4 or IL-6 expression^14^. In addition, rodenticide exposure was linked with significant elevations in total lymphocyte count^14^, whereas we found no evidence of lymphocytosis in our brodifacoum-exposed group. These contrasting findings may indicate innate differences between domestic cats and wild felids, or may allude to differences in the underlying etiology of immune dysfunction in bobcats compared to the cats in our study.

While few studies have investigated the pathogenesis of notoedric mange, sarcoptic mange – a closely related disease caused by *Sarcoptes scabeii* – is better described in the literature. In humans and animal models, severe crusting sarcoptic mange is attributed to an ineffective Th2 immune response^16,32^. Individuals who develop the severe form of the disease have marked numbers of CD8+ cells infiltrating the dermis and profound elevations of IL-4^16^. Numerous other cytokines (IL-2, TGF-β, IL-13) appear to be differentially affected^16,32–34^. Moreover, mites themselves secrete factors that alter cytokine production at the skin surface^16,35^. Interestingly, epicutaneous administration of warfarin in rats was found to induce the expression of pro-inflammatory cytokines (TNF-α, IL-17, and IL-1β) from skin explants and epidermal cells^36^, and had differential effects on granulocyte and lymphocyte proliferation in a whole-animal model^37^. While gross measures of immune function measured in our cats were not significantly altered – and those alterations that were detected were transient – brodifacoum exposure may subtly alter the Th1/Th2 immune balance at the cytokine level, which could affect feline immunity to external parasites. In addition, it is possible that rodenticide-mediated immune perturbations manifest locally in the dermis and thus were not detected via our experimental methods. However, the DTH responses of cats in our study indicate that skin-level immunity in brodifacoum-treated cats is predominantly normal.

Numerous other factors may affect the overall health and immune competence of wildlife species, including infectious diseases, stressors associated with living in urban environments, and exposure to environmental contaminants. While infectious diseases are known to circulate in wild felid populations and have well-described immunosuppressive effects^38–40^, Foley *et al*. report that unpublished data on disease testing in mange-infested bobcats has not shown any association between other infectious diseases and occurrence of generalize mange^17^.

Peri-urban wildlife demonstrate glucocorticoid alterations in response to a variety of urban factors, including noise pollution, light pollution, and changes to normal diets^41,42^. Bhattacharjee *et al*. have described elevated levels of fecal cortisol in tigers living adjacent to anthropogenic activities^43^, and numerous studies have shown that persistently elevated glucocorticoid levels impact immune function in various species^44–47^. Thus, stress-associated immune suppression may be an important factor in the development of disease in wildlife.

Beyond exposure to anticoagulant rodenticides, animals living in urban or semi-urban areas are widely exposed to a variety of potentially bioactive contaminants that leach into the environment, including polyaromatic hydrocarbons, organophosphates, and organochlorines^48–50^. These contaminants of concern (COC) have well-documented effects on the immune function of numerous animal species^51–53^, and are believed to bioaccumulate in upper-level predators^53–56^. Studies in laboratory animals and wildlife have indicated that these compounds cause measurable immune system derangements^49,56–60^. Thus, these agents must also be considered and investigated as potential auxiliary, potentiating, or primary factors driving immune derangements in wildlife species.

Collectively, there are most likely numerous factors that contribute to immune modulation in wild felids, each of which warrants further investigation. Importantly, our study demonstrates that healthy domestic cats do not manifest overt signs of immune dysregulation despite chronic exposure to environmentally relevant, sub-toxic doses of brodifacoum. There may be specific windows of time when certain immune responses are diminished in brodifacoum-exposed cats, but this appears to be transient. It is likely that comorbidities, concurrent stressors, exposure to environmental contaminants, and other variables may all play a role in the ultimate development of immune dysfunction in wild felids with evidence of anticoagulant rodenticide exposure, and that brodifacoum exposure is only one possible component of this phenomenon.

## Methods

### Animals

Specific-pathogen-free (SPF) domestic cats were randomly assigned to either a sham-bait or brodifacoum-treated group (n = 5/group). Cats were free from feline herpes virus, feline calicivirus, feline leukemia virus, feline immunodeficiency virus, feline panleukopenia virus, toxoplasmosis, *Bartonella henselae*, *Giardia* spp., *Cytoisospora* spp., and *Cryptosporidium* spp. Each group contained 3 neutered males and 2 intact females, all approximately 9–10 months of age. Cats were gang-housed according to treatment group and received a nutritionally complete feline diet and water *ad libitum*. All animal experiments were reviewed and approved by Colorado State University’s Institutional Animal Care and Use Committee and were carried out in accordance with relevant regulations and guidelines.

### Vaccinations and Brodifacoum Administration

All cats were subcutaneously vaccinated with 50 µg of ovalbumin (OVA, InvivoGen, San Diego, CA) dissolved in 1 ml sterile PBS on weeks -6, -4, and 2, and 50 µg of keyhole limpet hemocyanin (KLH, Sigma Aldrich, St. Louis, MO) dissolved in 1 ml PBS on weeks 2, 4, and 6. Cats in the brodifacoum-treated group were fed 0.05 mg/kg brodifacoum mixed into canned cat food once weekly from weeks 0 to 5. Cats in the control group received an equivalent amount of inert bait. Cats were fed individually during bait exposure and each cat consumed the full amount of bait at each dosing.

### Blood Collection and Tissue Collection

CBCs and prothrombin times (PTs) were measured twice weekly on weeks 0–6, and once weekly through week 10 (SCA2000 Veterinary Coagulation Analyzer, Synbiotics, Kansas City, KS) (IDEXX Coag Dx* PT cartridges, IDEXX Veterinary Diagnostics, Westbrook, ME). Baseline CBCs and PTs were collected prior to initial bait exposure. Serum, plasma, whole blood, and leukocytes were saved from each blood collection and stored at −80 °C for analysis at later time points. Six weeks following final bait exposure (week 11), cats were anesthetized for a laparotomy to obtain a 1–3 g section of liver for analysis of brodifacoum levels.

### Whole Blood and Liver Brodifacoum Levels

Whole blood from weeks 1–10 and liver biopsies from week 11 from both brodifacoum- and sham-bait-treated cats were measured for brodifacoum concentrations using high-performance liquid chromatography combined with atmospheric pressure chemical ionization and tandem mass spectrometry (HPLC-APCI-MS/MS). Liver and blood from non-treated cats acted as negative controls. Whole blood (0.2 mL) was extracted with a water-acetonitrile mixture, phase separated with excess NaCl, and the acetonitrile phase analyzed by HPLC-APCI-MS/MS. Liver (~0.2 g) was extracted the same way, except that an additional clean-up step with dispersive solid-phase extraction (C18 and primary-secondary amine sorbents) was performed prior to analysis. Method accuracy using control cat liver fortified with 0.028 to 2.3 µg/g brodifacoum (n = 12) ranged from 90% to 101%. Control whole cat blood fortified with 0.0058 to 0.47 µg/mL brodifacoum (n = 24) ranged from 87% to 128%. Brodifacoum detection and quantitation limits estimated from 3X and 10X signal-to-noise ratio were 0.006 and 0.019 µg/g in liver; 0.0007 and 0.0022 µg/mL in whole blood.

### Delayed-type Hypersensitivity (DTH) Reaction

**Figure 10:**
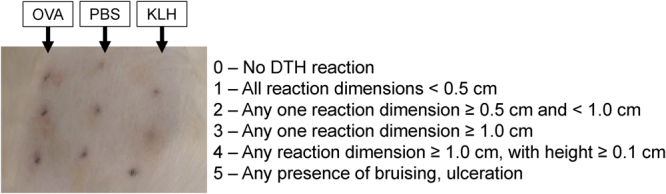
Left panel depicts typical delayed-type hypersensitivity (DTH) reaction appearance on dorsum of cat. Triplicate series of each antigen were injected (0.1 ml/injection). From left to right: ovalbumin (OVA), PBS, keyhole limpet hemocyanin (KLH). Right panel shows DTH scoring algorithm, with each reaction scored from 0–5. Mean DTH score was calculated for each antigen for each cat.

Cell-mediated immunity to OVA and KLH were tested with delayed-type hypersensitivity (DTH) tests on weeks -3, 2, 4, 6, and 9. Briefly, 0.1 ml of OVA (1 mg/ml), KLH (1 mg/ml), and sterile PBS were injected intradermally in triplicate on the shaved dorsum of each cat. Degree of induration at each site was measured with calipers at 24, 48, and 72 h post-injection. The concentration of OVA was decreased from 1 mg/ml to 0.25 mg/ml for the final two DTH tests in both groups due to exuberant induration in vaccinated cats. A scoring algorithm was used to determine the mean induration for each antigen (Fig. 10) and unpaired t-tests with Holm-Sidak correction for multiple comparisons were used to test for significant differences between the treatment and control group means at each time point. Analyses were conducted using GraphPad Prism 6.0 software (La Jolla, CA) and p-values < 0.05 were considered significant.

### Anti-OVA and Anti-KLH ELISA

Anti-OVA and anti-KLH antibodies were quantified in the serum of treated and untreated cats collected on weeks 0, 4, 5, 6, 7, and 10. ELISA plates were prepared by adsorbing OVA and KLH to Immulon H2B high binding plates (Thermo Scientific, Rochester, NY) for 16–18 h at 4 °C. Each well was coated with 100 µl of antigen diluted in 50 mM carbonate buffer at a concentration of 0.5 ng/µl. Plates were blocked with 300 µl/well of wash buffer (2 mM imidazole, 160 mM NaCl, and 0.5 mM EDTA) containing 2% bovine serum albumin (BSA).

Serial two-fold dilutions of serum from each cat were added to 96-well plates (100 µl/well) and incubated for 2 h at room temperature on an orbital shaker. Supernatants were discarded, and wells were washed 5 times using an automatic plate washer (EL_X_50 AutoStrip Washer, Bio-Tek Instruments, Inc.) and wash buffer with 0.2% Tween 20. Goat anti-cat IgG (H+L) peroxidase conjugate (Cappel Research Reagents, MP Biomedicals, LLC, Solon, OH) was diluted 1:5000 in ELISA diluent with 5% normal mouse serum (Invitrogen, Rockford IL) and 100 µl was added to each well. Plates were incubated at room temperature on an orbital shaker for 1 h. Supernatants were discarded, and wells were washed 5 times using an automatic plate washer as before.

One hundred µl of TMB Microwell Peroxidase Substrate System (KPL, Gaithersburg, MD) was added to each well. Plates were incubated at room temperature on an orbital shaker for approximately 10 min. The peroxidase reaction was stopped by addition of 50 µl of 2.5 N H_2_SO_4_.

Plates were analyzed on a MultiSkan Spectrum ELISA plate reader (Thermo Labsystems) at 450 nm. A positive cutoff was set at twice the absorbance of the average reading of all negative serum samples on the plate. Pooled serum from 4 vaccinated cats served as a positive control. Buffer-only and pooled serum from unvaccinated cats were used as negative controls. Group means were compared using unpaired t-tests with Holm-Sidak correction for multiple comparisons to test for significant differences between the treatment and control group titers at each time point (GraphPad Prism 6.0). P-values < 0.05 were considered significant.

### IgE ELISA

Abcam’s IgE Cat ELISA Kit (Abcam, Cambridge, MA) was used to determine the concentration of serum IgE from each cat on weeks 0, 4, and 6 according to manufacturer’s instructions. Briefly, serum was diluted 1:50 in 1x IgE Cat Diluent. One hundred µL of standards 1–6, a blank control, and diluted samples were added in duplicate to the plate and incubated for 1 h. Following incubation, well contents were discarded and wells were washed 4 times with 1x Wash Buffer. After final wash, well contents were discarded and 100 µL of 1x IgE Cat HRP Conjugate was added to each well and incubated for 1 h in the dark. Following incubation, wells were again washed 4 times with 1x Wash Buffer. 100 µL of TMB Substrate Solution was then added to each well and incubated for 10 min, followed by the addition of 100 µL Stop Solution to each well. Absorbance at 450 nm was measured using a MultiSkan Spectrum ELISA plate reader and mean concentration of IgE was calculated relative to the standards. Repeated measures ANOVA with multiple comparisons was used to evaluate differences in IgE concentration between treatment groups over time and at individual time points. Analyses were conducted using GraphPad Prism 6.0. P-values < 0.05 were considered significant.

### Cell Proliferation Assay

Peripheral blood mononuclear cells (PBMCs) were isolated from each cat’s blood using a standard Histopaque (Sigma, St. Louis, MO) isolation method^61^. Red blood cells were removed using Ammonium-Chloride-Potassium lysing buffer. PBMCs (2.5 × 10^5^) from each cat were added to each of 12 wells in a 96-well tissue-culture plate with culture medium (Dulbecco’s modified Eagle’s medium (DMEM) with GlutaMAX-1, 10% fetal bovine serum (FBS), and 1x penicillin-streptomycin (10,000 U/L penicillin and 10,000 μg/L streptomycin), and 1 μg/ml of amphotericin B).

Cells were stimulated with concanavalin A (ConA), OVA, or KLH at concentrations of 10 µg/ml, 100 µg/ml, and 100 µg/ml, respectively, or were inoculated with culture medium alone. Stimulated cells were incubated at 37 °C for 72 h (ConA) or 96 h (OVA, KLH, unstimulated cells). Cells used for single staining were stimulated with ConA or medium only. Each cat’s cells were run in triplicate for each treatment.

After incubation, cells were pulsed with 1 µM 5-ethynyl-2′-deoxyuridine (EdU) for 24 h at 37 °C. One hundred µL of supernatant was collected from each well and frozen at −80 °C for cytokine analysis (see below). Cells were washed with FACS buffer and blocked with normal mouse serum. Antibodies were labeled using Abcam’s APC/Cy7 Conjugation kit (Cambridge, MA) (Mouse Anti-Feline CD5) according to kit instructions. Antibodies were diluted in FACS buffer (CD5 APC-CY7 1:750), added to cells at 50 µl/well, and incubated at room temperature in the dark for 30 min. Cells were then washed, fixed with 4% paraformaldehyde for 10 min, and then washed again with FACS buffer. Antibody single staining was accomplished similarly by addition of only 1 antibody per set of 3 ConA and 3 unstimulated wells. Antibody single stains underwent the EdU detection protocol without the addition of the EdU Alexa Fluor. The EdU single stain was accomplished by not surface staining with any antibodies and only performing EdU detection.

The Click-iT Plus EdU Alexa Fluor 647 Flow Cytometry Assay Kit (Life Technologies Corporation, Carlsbad, CA) was used to detect cell proliferation. Manufacturer’s protocols were followed with modifications to run smaller reaction volumes (60 µl total volume) per well in a 96-well flow cytometry plate. Reagent relative concentrations were retained.

**Figure 11:**
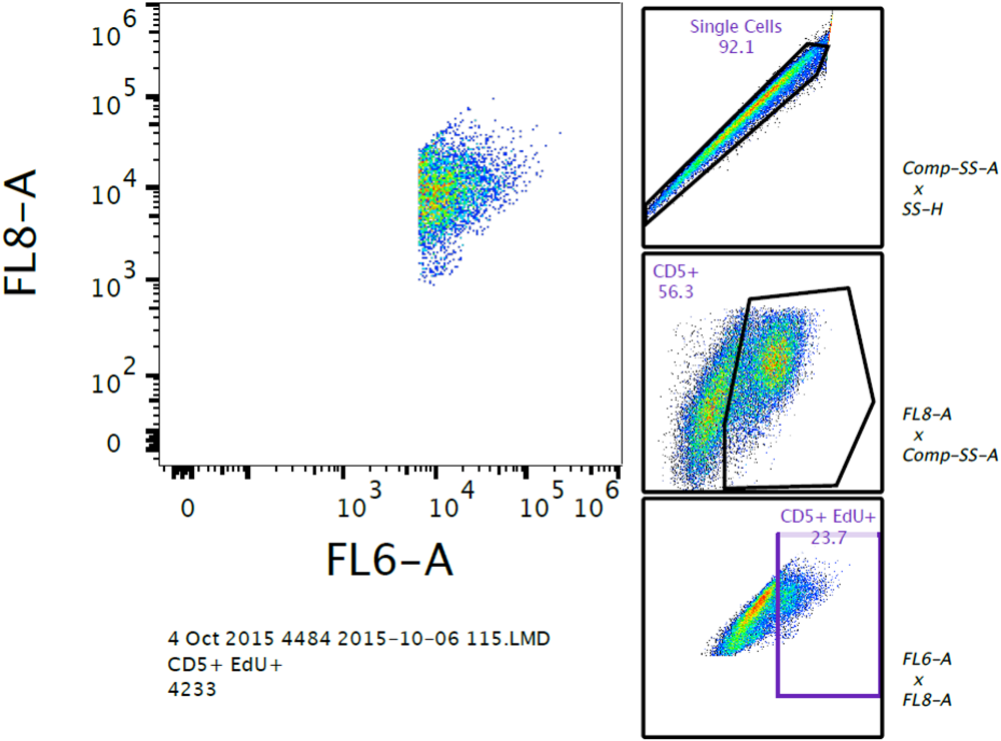
Gating tree used to identify CD5+/5-ethynyl-2′-deoxyuridine (EdU)+ cells. Single cells were identified by plotting side scatter area by side scatter height. Cells aligning linearly were counted as single cells. CD5+ cells were identified from single cells by plotting FL8 (the channel recognizing APC-Cy7) by side scatter area. Finally, CD5+ EdU+ cells were identified from CD5+ cells by plotting FL6 (the channel that detects APC) by FL8.

Stained cells were re-suspended in FACS buffer and run on a Beckman Coulter Gallios Flow Cytometer (Beckman Coulter, Brea, CA). All results were based on cell counts of 10,000 or more. List mode-files were analyzed using FlowJo software (Ashland, OR). Gating was achieved as diagrammed in Fig. 11. Compensation was based on single color staining of cells undergoing identical treatments as those being analyzed for each of the fluorescently-labeled antibodies used. Unpaired t-tests with Holm-Sidak correction for multiple comparisons were used to evaluate differences in cytokine concentration between treatment groups. Analyses were conducted using GraphPad Prism 6.0 software and p-values < 0.05 were considered significant.

### Quantification of cytokine expression in PBMCs during brodifacoum treatment

Cytokine concentrations in unstimulated PBMCs and ConA-stimulated PBMCs were measured using a commercially available MILLIPLEX® MAP Feline Cytokine/Chemokine Magnetic Bead Panel (fluorophore-conjugated microspheres, Millipore) per manufacturer’s instructions. Briefly, 25 µl from each triplicate sample was combined into one sample per animal. Fifty µl of each combined sample was incubated with a composite panel of microspheres coupled with capture antibodies to INFγ, IL-1β, IL-2, IL-8, IL-10, MCP-1, TNFα, Fas, SDF-1, SCF, RANTES, PDGF-BB, KC, IL-18, IL-13, IL-12 (p40), IL-6, IL-4, GM-CSF, and Flt-3 ligand. Following incubation with biotinylated secondary antibodies and streptavidin-conjugated phycoerythrin (PE), soluble cytokine molecules were detected in each sample using a Luminex® 200™ detection system. Final analyte concentration was calculated using manufacturer-provided standard curves for each analyte and Bio-Plex™ Manager 5.0 software (Bio-Rad). Repeated measures ANOVA with multiple comparisons was used to evaluate differences in cytokine concentration among treatment groups over time and at individual time points. Analyses were conducted using GraphPad Prism 6.0 software and p-values < 0.05 were considered significant.

### PIVKA Assay

Proteins Induced by Vitamin K Antagonism or Absence (PIVKA) were measured using a commercially available human quantitative sandwich enzyme immunoassay (MyBioSource, San Diego, CA). Plasma samples from all cats from weeks 0 and 9 were diluted 1:4 and applied to duplicate wells. The PIVKA II standard (0–20 ng/ml) was also applied in duplicate. Bound PIVKA is detected using a peroxidase-labeled antibody and peroxidase substrate. Optical density (OD) of each sample, measured at 450 nm, was used to calculate the quantity of PIVKA II in the feline plasma samples using a line generated from the OD of the standards. Two canine patient plasma samples were used as negative and positive controls. The negative control sample was from a dog with a normal coagulation profile and no known exposure to vitamin K antagonists. The positive control sample was collected as part of a clinical diagnostic procedure from a dog with known warfarin administration, and a prolonged international normalized ratio (INR). The positive control sample contained 0.3 ng/ml of PIVKA II.

### Factor X Assay

Plasma collected from all cats on weeks 0 and 9 was prepared using the DiaPharma Factor X Kit (DiaPharma Group, Inc., West Chester, OH) following manufacturer’s instructions for the quantitative determination of factor X activity. Russell’s Viper Venom (RVV) specifically activates factor X to factor Xa, and factor Xa cleaves a chromogenic substrate to produce a color change. Optical density (OD) of the reaction well is directly proportional to the factor X activity in plasma. A standard curve was calculated using laboratory-prepared canine pooled plasma in serial two-fold dilutions representing factor X activities of 50% to 0.7815%. Logarithmic transformation of the percent activity was then used to generate a linear equation with optical density. Samples were diluted 1:4 and then applied in duplicate to a 96-well plate and warmed to 37 °C for 3–4 min. Factor Xa chromogenic substrate was added followed by RVV and calcium dichloride. The plate was then incubated for an additional 3 min for color development and 20% acetic acid was added to stop the color change. The ODs were measured at 405 nm using a plate reader (Syndergy H1, BioTek). Sample means were used to determine activity based on the equation for the standard curve.

### Data Availability

The datasets generated and/or analyzed during the current study are available from the corresponding author on reasonable request.

## Electronic supplementary material

Supplementary Information
