# Supplementary material for: Effects of Low-level Brodifacoum Exposure on the Feline Immune Response
Source: Sci Rep. 2018 May 25;8:8168. doi: 10.1038/s41598-018-26558-3 (PMC5970145; doi:10.1038/s41598-018-26558-3)
Supplement: Supplementary file 1 — Supplementary Information [file 41598_2018_26558_MOESM1_ESM.pdf]

Supplementary Information

Effects of Low-level Brodifacoum Exposure on the Feline Immune Response.

Jennifer H. Kopanke, Katherine E. Horak, Esther Musselman, Craig A. Miller, Kristine Bennett, Christine S. Olver, Steven F. Volker, Sue VandeWoude, and Sarah N. Bevins

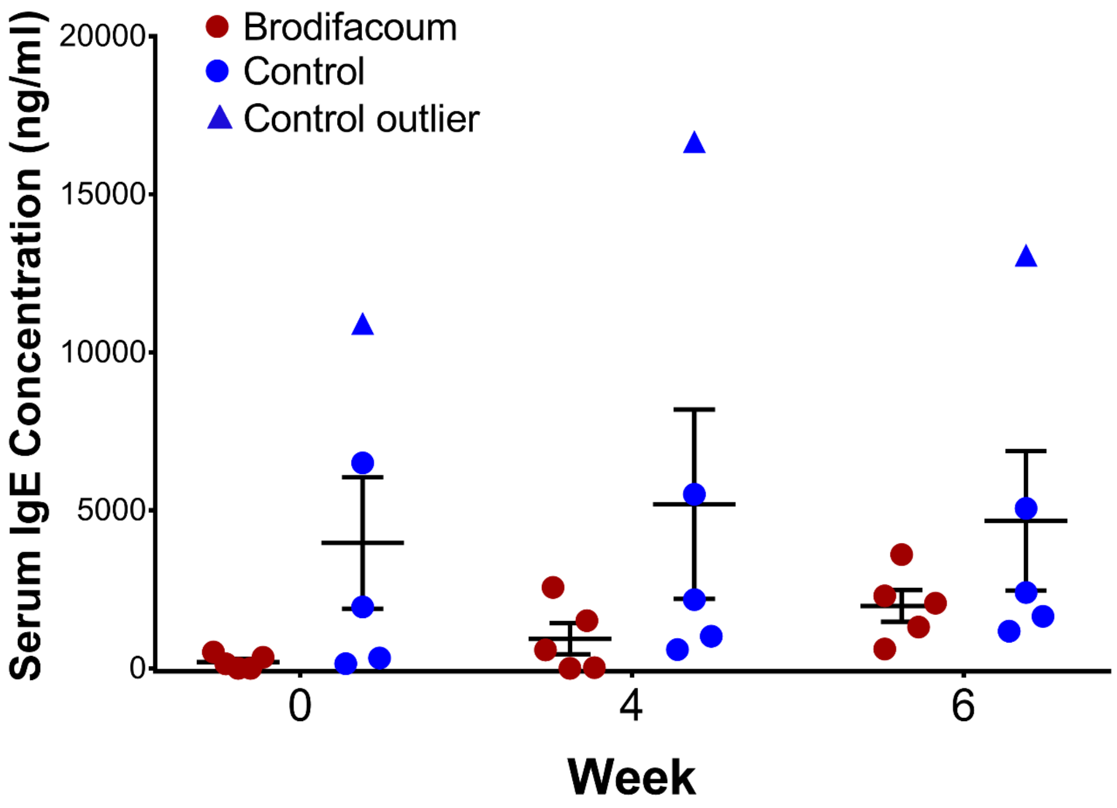

Supplementary Figure S1. Serum IgE concentrations determined via commercial feline IgE ELISA kit show that cats in the control group have higher levels of IgE than cats in the brodifacoum-treated group. This difference existed prior to brodifacoum treatment (week 0) and was heavily influenced by the presence of one outlier cat in the control group, which is depicted by ▲. Error bars show the mean and standard deviation at each time point.
